# The Role and Mechanism of *α*-Klotho in the Calcification of Rat Aortic Vascular Smooth Muscle Cells

**DOI:** 10.1155/2015/194362

**Published:** 2015-11-02

**Authors:** Tianlei Chen, Huijuan Mao, Cheng Chen, Lin Wu, Ningning Wang, Xiufen Zhao, Jun Qian, Changying Xing

**Affiliations:** ^1^Department of Nephrology, Jiangsu Province Hospital, The First Affiliated Hospital of Nanjing Medical University, Nanjing 210029, China; ^2^Department of Nephrology, The First People's Hospital of Changzhou, The Third Affiliated Hospital of Soochow University, Changzhou 213000, China

## Abstract

*Objective*. To investigate the role and possible mechanism of *α*-Klotho in the calcification and the osteogenic transition of cultured VSMCs.* Methods*. VSMCs were cultured* in vitro* and divided into 5 groups, each using a different medium: (1) control; (2) *β*-GP; (3) *β*-GP + Klotho; (4) *β*-GP + LiCl; (5) *β*-GP + Klotho + LiCl. Calcium deposits were visualized using Alizarin Red S staining. The calcium concentrations were determined by the o-cresolphthalein complexone method. BMP2, Runx2 and *β*-catenin levels were estimated by western blotting, and the level of *α*-SMA was determined by using immunofluorescence at day 12.* Results*. *β*-GP induced an increase in the expression of BMP2, Runx2, and *β*-catenin. The calcium content increased, and the expression of *α*-SMA decreased. Alizarin Red S staining was positive under the high phosphorus conditions. BMP2, Runx2, and *β*-catenin levels and the calcium content decreased when the cells were cultured with rmKlotho; however, the levels of each were upregulated after treatment with the LiCl.* Conclusions*. Klotho can ameliorate the calcification and osteogenic transition of VSMCs induced by *β*-GP. The mechanism of Klotho in preventing calcification in VSMCs may be partially mediated by the inhibition of the Wnt/*β*-catenin signaling pathway.

## 1. Introduction

The Klotho protein was originally identified as an aging suppressor [1], and it has a key role in the metabolism of minerals. The balance of calcium and phosphorus in the blood, bones, and cerebrospinal fluid of all vertebrates is appropriately adjusted by the Klotho protein. The levels of Klotho in the blood and urine of chronic kidney disease (CKD) patients are significantly reduced compared with the general population, and CKD patients have a high incidence of vascular calcification [2].* Klotho* gene knockdown mice show obvious vascular calcification and premature aging [3], and the overexpression of the Klotho gene in mice has the completely opposite results [4]. The Klotho protein significantly reduces the Na-Pi cotransporter activity of vascular smooth muscle cells (VSMCs) [5]. Altogether, these data indicate that Klotho may play a crucial role in antivascular calcification. However, some researchers have reported that Klotho has no effect on the calcification of human and mouse VSMCs [6, 7]. The effect of Klotho on the calcification of VSMCs is highly controversial, and its mechanism is not well known. In this study, recombinant mouse Klotho (rmKlotho) was directly applied to VSMCs that were stimulated by *β*-glycerophosphate (*β*-GP) to investigate the role and possible mechanism of Klotho in the calcification and osteogenic transition of cultured rat aortic VSMCs.

## 2. Methods

### 2.1. Culture of Rat VSMCs

The A7r5 cell line, originally derived from embryonic rat aorta, was purchased from the American Type Culture Collection (ATCC, Manassas, VA). The cells were cultured in Dulbecco modified Eagle's medium (DMEM) containing 10% fetal bovine serum (FBS, Gibco), 100 U/mL penicillin, and 100 *μ*g/mL streptomycin and maintained in an incubator at 37°C in a humidified 5% CO_2_ atmosphere.

### 2.2. The Induction of Calcification and the Grouping of the Cells

At 70 to 80% confluence, the culture medium was replaced with serum-free medium for 24 h to render the cells quiescent. Calcification was induced by adding medium with 10 Mm *β*-GP (Sigma-Aldrich, USA), 50 *μ*g/mL vitamin C, and 1 × 10^−7^ mol/L insulin for 12 d. The VSMCs were divided into 5 groups: (1) the control group, which was cultured in ordinary medium; (2) the calcification group (*β*-GP group), which was cultured in medium with 10 mM *β*-GP added; (3) the Klotho intervention group (*β*-GP + rmKlotho group), which was cultured in medium with *β*-GP as in group (2) and different concentrations (25, 50, 100, and 200 ng/mL) of rmKlotho (1819-KL, R&D, Minneapolis, MN) to choose an appropriate treatment concentration for rmKlotho; (4) the LiCl intervention group (*β*-GP + LiCl group), which was cultured in medium with *β*-GP as in group (2) and the Wnt signaling agonist LiCl (5 mM, Amresco, USA); and (5) the Klotho + LiCl intervention group (*β*-GP + rmKlotho + LiCl group), which was cultured in medium that contained *β*-GP as in group (2), rmKlotho (50 ng/mL), and LiCl (5 mM). rmKlotho was added at each culture medium replacement.

### 2.3. Assessment of the Calcification of VSMCs

After VSMCs were cultured for 12 d, the calcium concentrations of the cell layers were determined by the o-cresolphthalein complexone method and normalized to protein content as previously described [8]. Alizarin Red S staining was used to detect calcium deposition. Cells were rinsed in phosphate buffered saline (PBS, Invitrogen) and fixed in 95% ice-cold ethanol prior to staining with 2% Alizarin Red S (pH 4.2, Sigma) for 15 min at room temperature (RT). The emergence of the mineralized nodules was considered as positive.

### 2.4. *α*-SMA Immunofluorescence

The cells were washed and fixed with ice-cold paraformaldehyde before blocking with 3% bovine serum albumin (BSA). Samples were then treated at 37°C for 1 h with the rabbit monoclonal anti *α*-SMA antibody (1 : 100; Boster, Wuhan, China), followed with three PBS washes before using the FITC-conjugated AffiniPure goat anti-rabbit IgG(H+L) antibody (1 : 100; EarthOx, USA) for detection. Labeled cells were examined under a Zeiss confocal microscope, and images were obtained with the laser scanning confocal microscope LEICA TCS SP2 (Leica, Wetzlar, Germany). The images were then quantified by Image J.

### 2.5. Western Blot Analysis of BMP2, Runx2, and *β*-Catenin

After the proteins were isolated from each group of cells, the proteins were transferred to membranes, and the membranes were incubated overnight at 4°C with rabbit anti-rat BMP2 (1 : 1000; Abcam, UK), Runx2 (1 : 500; Proteintech), and *β*-catenin (1 : 1000; Proteintech) polyclonal antibodies. After the membranes were washed thrice with Tris buffer containing 0.1% Tween-20 (TBST), they were incubated with goat anti-rabbit IgG-HRP (1 : 5000; ZSGB-BIO, Beijing). Bands were visualized with a chemiluminescence system (ChemiDoc XRS, Bio-Rad, PA, USA). The bands on the films were quantified using the Quantity One software (Bio-Rad, CA, USA). The BMP2, Runx2, and *β*-catenin levels were normalized to the levels of GAPDH as an internal control.

### 2.6. Analysis of Experimental Data

Statistical analyses were performed using SPSS software v19.0 (SPSS, Chicago, IL). The data were expressed as the means ± standard deviations, and the comparison among groups was performed using *t*-tests. *P* < 0.05 indicated a significant difference.

## 3. Results

### 3.1. Calcification of VSMCs Induced by *β*-GP

**Figure 1:**
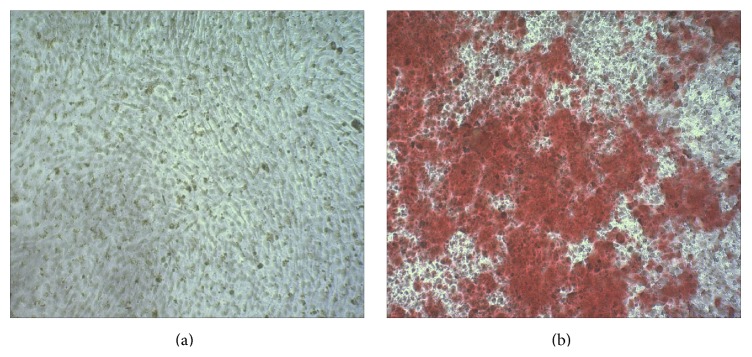
Alizarin Red S (2%, pH 4.2) staining was performed on cells incubated under different medium for 12 d. (a) Control group. (b) *β*-GP group.

**Figure 2:**
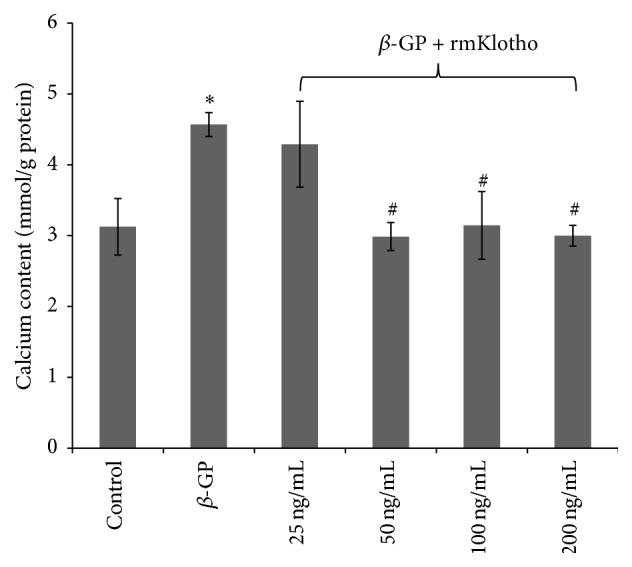
The calcium content of the extracellular matrix. From left to right: control, *β*-GP, *β*-GP + 25 ng/mL Klotho, *β*-GP + 50 ng/mL Klotho, *β*-GP + 100 ng/mL Klotho, and *β*-GP + 200 ng/mL Klotho group. The quantification of the calcium content is represented as the mean with the standard error (SE) (*n* = 5 per group) for each group in its respective column. ^*∗*^ *P* < 0.01* versus control group*; ^#^ *P* < 0.01* versus β-GP group*.

Briefly, 10 mM *β*-GP induced VSMC calcification. In the *β*-GP-treated group, the extracellular matrix contained significant calcification crystallization, and the Alizarin Red S staining was positive for calcium deposits (Figure 1). The calcium content of the extracellular matrix in the *β*-GP-treated group was significantly increased compared with the control group after 12 d incubation (Figure 2).

### 3.2. The Role of rmKlotho in the Calcification of VSMCs

50–200 ng/mL rmKlotho can equally reduce the calcium content of the extracellular matrix. Here, 50 ng/mL was selected as the most suitable intervention concentration of rmKlotho for the following studies (Figures 2 and 5).

### 3.3. The Role of rmKlotho in the Osteoblastic Differentiation of VSMCs

**Figure 3:**
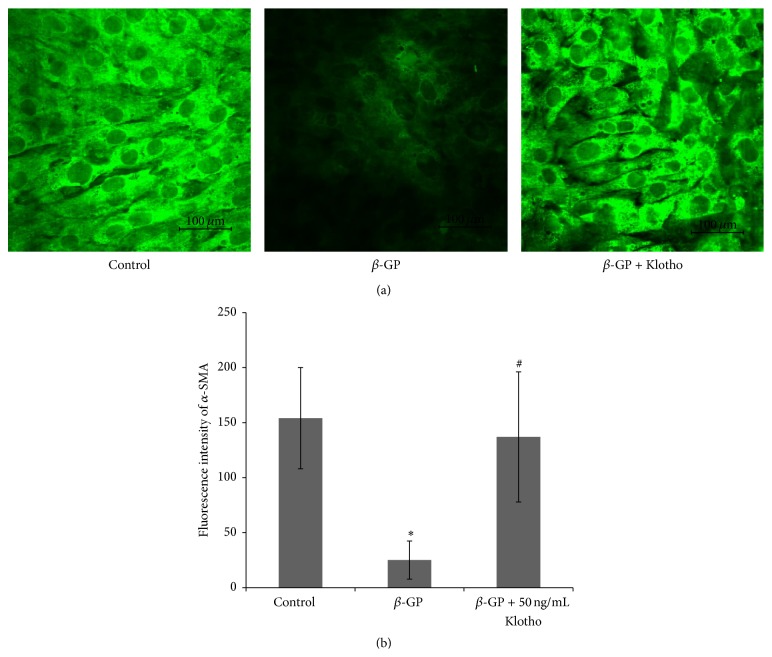
*α*-SMA was downregulated during the calcification of VSMCs. VSMCs were treated with or without 10 mM *β*-GP and with or without 50 ng/mL Klotho for 12 d. (a) Representative *α*-SMA expression levels from VSMCs following treatment with control medium, *β*-GP medium, or *β*-GP + 50 ng/mL rmKlotho medium for 12 days. (b) The quantification of *α*-SMA is represented as the mean with the SE (*n* = 5 per group) for each group in its respective column shown in (b). ^*∗*^ *P* < 0.01 versus* control group*; ^#^ *P* < 0.01 versus *β-GP group*. Scale bar = 100 *μ*m.

**Figure 4:**
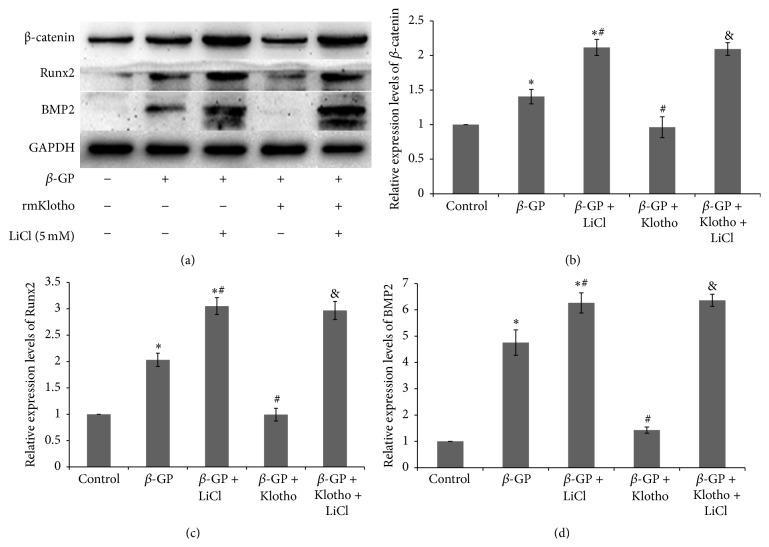
(a) Representative BMP2, Runx2, and *β*-catenin expression levels from VSMCs following treatment with the control, *β*-GP, *β*-GP + LiCl, *β*-GP + rmKlotho, or *β*-GP + rmKlotho + LiCl media for 12 d. BMP2, Runx2, and *β*-catenin expression was analyzed by western blotting using GAPDH as the loading control. The quantification of the expression levels of (b) *β*-catenin, (c) Runx2, and (d) BMP2 is represented as the mean with the SE (*n* = 5 per group) for each group in its respective column. ^*∗*^ *P* < 0.01* versus control group,* ^#^ *P* < 0.01* versus β-GP group*, and ^&^ *P* < 0.01* versus β-GP + Klotho group.*

To further investigate the role of Klotho in the osteoblastic differentiation of VSMCs, the expression levels of *α*-SMA, BMP2, and Runx2 were determined in the control and treated groups. The immunofluorescence intensity of *α*-SMA markedly decreased in the *β*-GP treatment group compared with the intensity of the control group; however, the intensity was enhanced following treatment with rmKlotho at 50 ng/mL (Figure 3). Compared with the control group, the levels of BMP2 and Runx2 increased significantly in the *β*-GP-treated group, while rmKlotho treatment markedly reduced the expression of these two proteins (Figure 4).

### 3.4. Klotho and the Wnt/*β*-Catenin Pathway

**Figure 5:**
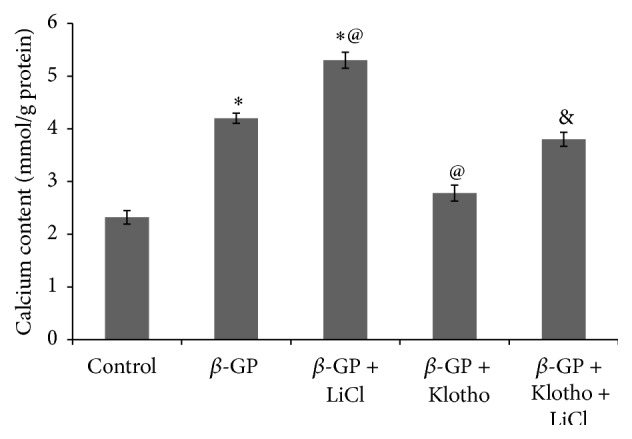
Calcium content in the extracellular matrix of the VSMCs following treatment with the control, *β*-GP, *β*-GP + LiCl, *β*-GP + rmKlotho, or *β*-GP + rmKlotho + LiCl medium for 12 d. The quantification of calcium content is represented as the mean with the SE (*n* = 5 per group) for each group in its respective column. ^*∗*^ *P* < 0.01 versus* control group*, ^@^ *P* < 0.01 versus *β-GP group*, and ^&^ *P* < 0.01 versus *β-GP + Klotho group*.

The relative expression level of *β*-catenin was increased in the *β*-GP-treated group compared with the control group. The level of expression of *β*-catenin, BMP2, and Runx2 as well as the amount of calcium deposited decreased in the *β*-GP + rmKlotho-treated group compared with the *β*-GP-treated group (Figures 4 and 5). LiCl, which is an agonist of the Wnt/*β*-catenin pathway, significantly decreased *β*-catenin, BMP2, and Runx2 expression as well as calcium levels compared with the *β*-GP-treated group at d 12 of incubation (*β-GP + LiCl group*  versus *β-GP group*). Furthermore, the levels of *β*-catenin, BMP2, and Runx2 as well as the calcium content were all increased in the Klotho + LiCl treatment group compared with the Klotho only-treated group (*β-GP + Klotho + LiCl group* versus *β-GP + Klotho group*) (Figures 4 and 5).

## 4. Discussion

Vascular calcification is a common pathological change found in the elderly and in atherosclerosis, hypertension, and diabetes patients. This condition can increase the risk of myocardial infarction and death, especially in patients with CKD [9]. VSMCs can transdifferentiate into a synthetic/osteoblast-like phenotype, which is induced by metabolism imbalance and uremia, thus promoting the occurrence of vascular calcification [10, 11]. Previous studies [12] have suggested that phosphorus can induce both Runx2 and osterix expression. Elevated phosphorus concentrations in the cell by the sodium/phosphate cotransporter Pit-1 may promote osteoblast differentiation and vascular calcification* in vivo*. Serum phosphate levels are important to determine calcification levels, and reducing serum phosphate levels can eliminate soft tissue and vascular calcification, even in the presence of extremely high calcium and 1,25-dihydroxyvitamin D levels in the serum [13]. In our study, after treatment with 10 mM *β*-GP for 12 d, Alizarin Red S staining of VSMCs revealed calcified nodules. Additionally, with this treatment, the expression of BMP2 and Runx2 and the extracellular matrix calcium content significantly increased, whereas the level of *α*-SMA reduced, which was similar to that reported in previous research [5, 12].

Soluble Klotho is derived from the transmembrane protein after cleavage by secretases, which are predominantly expressed in distal convoluted tubule epithelial cells.* Klotho* knockout mice exhibit accelerated aging with widespread ectopic calcification, including vascular calcification [14]. In certain instances, CKD can be associated with decreased Klotho levels [3]. Controversially, a candidate gene study of the Framingham Offspring Cohort [6] did not exhibit an association between the functional KL-VS variant of Klotho and the presence of valvular or vascular calcification. Other studies [7] reported that FGF23 has no effect on phosphate uptake or phosphate-induced calcification, regardless of the phosphate concentration or even in the presence of soluble Klotho. Whether Klotho directly affects vascular calcification remains controversial.

In our research, VSMCs were cultured in medium containing 10 mM *β*-GP and different concentrations of Klotho. The results showed a significant reduction in the calcium content of the extracellular matrix when the concentration of Klotho was more than 50 ng/mL. Klotho (50 ng/mL) was used to treat VSMCs in subsequent experiments. As a result, the expression of *α*-SMA in the *β*-GP + Klotho-treated group was significantly increased compared with the *β*-GP-treated group; however, BMP2 and Runx2 exhibited downregulated expression patterns. These data demonstrate that Klotho inhibits the differentiation of VSMCs into osteoblasts and ameliorates VSMC calcification.

At present, the mechanism of the influence of Klotho on the calcification of VSMCs is unclear. Various studies [15, 16] have suggested that Klotho is involved in regulating calcium and phosphate metabolism and acts as a collaborative receptor of FGF23. However, other studies [7, 17–20] do not support the Klotho-mediated FGF23 effects on the vasculature. Intravenous delivery of FGF23 elicited an increase in the expression of Egr-1 in renal but not arterial vasculature, which is a marker of Klotho-dependent FGF23 signaling. The calcification of bovine VSMCs was not altered after treatment with FGF23 (0.125–2 ng/mL). These results demonstrated that FGF23-Klotho signaling is absent in mouse arteries [17]. Studies in humans revealed the same results [7]. Moreover, some studies [18] reported that Klotho acts as a type of hormone in blood circulation. By binding with cell-surface receptors, Klotho is involved in the inhibition of the insulin/IGF signaling pathway, playing a variety of physiological and pathophysiological roles in different organs. Studies [19] have also reported that Klotho acts as a *β*-glucuronidase, hydrolyzing steroid *β*-glucuronides, or represents a novel mechanism for the regulation of the activity of cell-surface glycoproteins [20].

Wnt signaling plays a key role in vascular calcification [21]. The activation of the Wnt/*β*-catenin signaling pathway may increase the expression of BMP2 and stimulate the osteoblast differentiation of VSMCs. Vascular calcification is part of the active process of the transformation of VSMCs into the osteoblasts. In this study, we attempted to investigate the relationship between Klotho and the Wnt/*β*-catenin signaling pathways. Wnt signaling pathways are divided into classical and nonclassical pathways. Wnt signaling can be highly activated by various mechanisms, and its main function is to inhibit the proteolysis of *β*-catenin, which is controlled by phosphorylation. Free *β*-catenin can enter the nucleus and activate the target genes of Wnt. Steady-state levels of Axin are very important, as this scaffolding protein initiates the formation of the *β*-catenin degradation complex [22]. As a GSK3 inhibitor, LiCl upregulates Runx2, and Wnt signal-mediated osteoblastogenesis also exists [9]. Moreover, BMP-2 injection increases aortic Msx2 expression and medial artery calcification* in vivo* [23]. Thus, BMP-Msx2-Wnt signaling contributes to ectopic medial artery calcification. Wnt/*β*-catenin signaling induces BMP expression, whereas BMPs induce Wnt expression [24], suggesting that both BMP and Wnt signaling may synergistically regulate each other in osteoblasts, possibly through an autocrine or paracrine loop. In addition, a study [25] involving stem cells demonstrated Klotho can act as a Wnt antagonist and immunoprecipitates with a number of Wnt isoforms, including Wnt1, Wnt3, Wnt4, and Wnt5a. In our study, *β*-GP exposure resulted in calcification and a marked increase in *β*-catenin (*β*-GP group), whereas the level of *β*-catenin significantly decreased and calcification was alleviated following Klotho treatment (*β*-GP + rmKlotho group). However, the expression of *β*-catenin was obviously upregulated again after treatment with 5 mM LiCl (*β*-GP + LiCl group). In addition, our results showed that the effects of Klotho in calcification protection could be reversed after treatment with a Wnt signaling agonist (*β*-GP + rmKlotho + LiCl group). These data demonstrated that Klotho may attenuate osteoblastic differentiation and the calcification of VSMCs by inhibiting the Wnt/*β*-catenin signaling pathway.

We proposed a possible Wnt/*β*-catenin-BMP axis of calcification regulation by Klotho. *β*-GP exposure triggered the Wnt/*β*-catenin pathway and inhibited the degradation of intracytoplasmic *β*-catenin. Thus, more free *β*-catenin can enter the nucleus and activate the BMP genes, which subsequently induces the high expression of BMP2. BMP2 stimulates the transcriptional activation of Runx2. In addition, Klotho downregulates the expression of *β*-catenin (Figure 4) and also causes the subsequent downstream inhibition of BMP2, thus downregulating Runx2 through the autocrine or paracrine loop.

## 5. Conclusion and Limitation

Klotho attenuates osteoblastic differentiation and the calcification of VSMCs induced by a high content of phosphorus. The effect of Klotho on calcification may be associated with the classic Wnt/*β*-catenin pathway.

Unfortunately, in this study, we cannot determine the relationship between Klotho and the Wnt receptor. Whether this process relates to the glucuronic acid activity of Klotho requires further study. Moreover, the role of the Wnt signaling pathways is broad and complex. Further research is required to confirm whether Klotho affects other pathological processes through this pathway. The Klotho/Wnt pathway may be a promising new therapeutic target to prevent the calcification of vasculature.
